# Novel Process Noise Model for GNSS Kalman Filter Based on Sensitivity Analysis of Covariance with Poor Satellite Geometry

**DOI:** 10.3390/s21186056

**Published:** 2021-09-09

**Authors:** Yoji Takayama, Takateru Urakubo, Hisashi Tamaki

**Affiliations:** 1 Furuno Electric Co., LTD., Nishinomiya 662-0934, Japan; 2 Department of Information Science, Graduate School of System Informatics, Kobe University, Kobe 657-8501, Japan; t.urakubo@silver.kobe-u.ac.jp (T.U.); tamaki@al.cs.kobe-u.ac.jp (H.T.)

**Keywords:** GNSS, Kalman filter, satellite geometry, process noise

## Abstract

One of the great unsolved GNSS problems is inaccuracy in urban canyons due to Non-Line-Of-Sight (NLOS) signal reception. Owing to several studies about the NLOS signal rejection method, almost all NLOS signals can be excluded from the calculation of the position. However, such precise NLOS rejection would make satellite geometry poor, especially in dense urban environments. This paper points out, through numerical simulations and theoretical analysis, that poor satellite geometry leads to unintentional performance degradation of the Kalman filter with a conventional technique to prevent filter divergence. The conventional technique is to bump up process noise covariance, and causes unnecessary inflation of estimation-error covariance when satellite geometry is poor. We propose a novel choice of process noise covariance based on satellite geometry that can reduce such unnecessary inflation. Numerical and experimental results demonstrate that performance improvement can be achieved by the choice of process noise covariance even for a poor satellite geometry.

## 1. Introduction

Global Navigation Satellite System (GNSS) is an absolute positioning sensor widely used in various applications. GNSS can determine the location of a receiver based on trilateration by using pseudo-ranges between GNSS satellites and a receiver. It is well-known that positioning performance is dramatically degraded because of Non-Line-Of-Sight (NLOS) signal reception in urban environments [1]. NLOS signal reception occurs where tall buildings block Line-Of-Sight (LOS) direct signals from satellites and the signals are received via reflection and/or diffraction. Pseudo-ranges obtained from NLOS signals have tendency to be positive outliers [2], because they are computed from the times of flight of the signals. We, therefore, have to reject NLOS signals from position calculation to improve positioning accuracy in urban environments.

Many researchers have developed NLOS signal rejection methods based on measurement residuals [3,4,5], fisheye-camera images [6,7], 3D building maps with ray-tracing [8,9], and so on. It is possible to reject NLOS signals precisely by applying these methods, and then positioning performance can be improved if a sufficient number of satellites are available. Unfortunately almost all signals in dense urban environments could be NLOS signals. The precise rejection of NLOS signals would greatly reduce the number of satellites available in position calculation, thus cause poor satellite geometry, that is, a biased distribution of visible satellites. Since accuracy of trilateration is highly dependent on the geometric locations of the satellites as seen by a receiver, poor satellite geometry may result in inaccurate positioning. The above mentioned studies have not sufficiently dealt with the problem of poor satellite geometry caused by NLOS rejection. Taking satellite geometry into account in addition to NLOS rejection might yield more accurate positioning.

The estimate of position usually can be obtained by using Kalman filter [10,11] under some assumptions about process model, measurement model, and noise statistics. However, the estimation by Kalman Filter may suffer from its divergence [12,13]. A typical example of filter divergence occurs when the assumptions are inconsistent with actual model. The estimation-error covariance becomes excessively small due to the model errors, the filter gain is therefore small, and eventually the filter begins to ignore new measurements. As a consequence, estimate errors will be biased or diverge. Since these model errors are in general unknown and unbounded, it is difficult to solve the divergence theoretically.

A conventional and simple technique [14] to prevent filter divergence is to bump up process noise covariance to cover the uncertainty of model errors. This technique is easy to implement, that is, we have only to add fictitious process noise covariance to its nominal value. Since this technique is largely heuristic, it is important to discuss its effect not only on divergence but also on filter performance.

In the application of the technique to GNSS positioning [4], bumping up process noise covariance inflates estimation-error covariance in the prediction step. The inflated covariance can be reduced by processing observations right after the inflation, if satellite geometry is good. Consequently, the size of estimation-error covariance is kept to be large enough to avoid the divergence, but not too large. However, if satellite geometry is poor, what would be happened? The inflation cannot be reduced in a certain direction according to the geometry, and the estimation-error covariance would become larger than expected. This unexpectedly large covariance would lead to inaccurate positioning.

A proper choice of the process noise covariance has been of interest to many researchers so far. Some filtering techniques have been developed to have an estimation-error covariance that is insensitive to changes in process noise covariance, through the steady-state analysis of estimation-error covariance [15,16]. Furthermore, also noise-adaptive estimation techniques have been proposed [17,18,19]. However, these techniques cannot handle with preventing the performance degradation caused by poor satellite geometry, because they do not take satellite geometry into account.

There are some approaches that can be used to avoid the performance degradation caused by poor satellite geometry. Constrained Kalman filter techniques can be utilized to mitigate the degradation [20,21]. The techniques automatically detect poor satellite geometry, and estimate only the state variables for which poor satellite geometry would not cause the degradation. However, it is difficult to choose a proper threshold for detecting poor satellite geometry in a heuristic manner. The positioning degradation can also be resolved by integrating other sensors such as IMU (Inertial Measurement Unit) and LiDAR (Light Detection and Ranging) device into GNSS Kalman filter [4]. The observations from the sensors can compensate for the lack of information due to poor satellite geometry. However, adding those sensors is often unacceptable in terms of cost and installation space.

The main contribution of this paper is twofold. First, we clarify the fundamental relationship between process noise and satellite geometry through theoretical analysis. The analysis shows that the inflation of estimation-error covariance by fictitious process noise can be unexpectedly large for poor satellite geometry. Secondly, from the theoretical results, we propose a novel way to choose process noise covariance based on satellite geometry in order to remove the unexpected inflation. Comparing with the approaches mentioned above, our approach is based on a theoretical sensitivity analysis, and does not require other additional sensors.

This paper is constructed as follows. Section 2 illustrates the problem of poor satellite geometry by using motivating examples. We describe theoretical analysis for Kalman filter to show the relationship between process noise covariance and satellite geometry in Section 3. We then introduce the extended Kalman filter for GNSS stationary positioning and derive a novel way to choose process noise covariance based on satellite geometry in Section 4. We present simulation and experimental results in Section 5 and Section 6. Section 7 concludes this paper.

## 2. Positioning from Poor Satellite Geometry

### 2.1. Poor Satellite Geometry Caused by NLOS Signal Rejection

**Figure 1:**
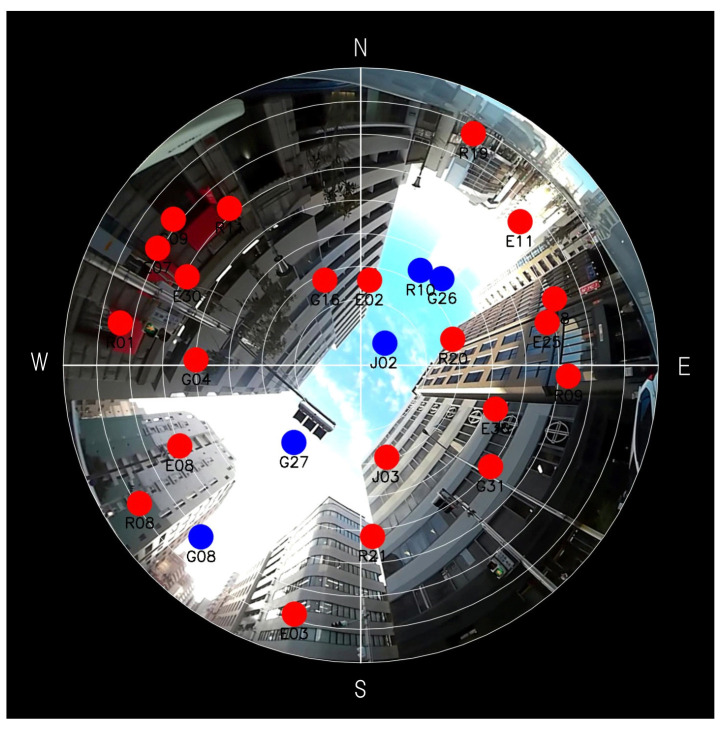
A sky plot onto a fisheye-camera image taken at Kobe city, Japan. A blue dot represents a LOS satellite and a red dot represents a NLOS satellite. N, S, E, and W stand for North, South, East, and West, respectively.

This section illustrates the problem caused by poor satellite geometry. Figure 1 is an example of a satellite sky plot superimposed on a fisheye-camera image taken at Kobe city, Japan. As shown in Figure 1, tall buildings surround the location and can block almost all direct signals from satellites. We suppose that satellites overlapped on buildings are NLOS satellites (denoted as red dots), and the others are LOS satellites (denoted as blue dots). If complete NLOS satellite rejection is done, only the five satellites will remain. We can see that remained satellites are located along the northeast direction. If we bump up process noise covariance to avoid the filter divergence, position uncertainty in the direction of southeast will become larger unintentionally. Although it will be explained from a theoretical point of view in Section 3, we will show it through a simulation study in this section.

### 2.2. Motivating Examples for Poor Satellite Geometry

In order to show the problem clearly, we present some simulation results using a simplified model for GNSS, under the assumption that five satellites are stationary and located in poor satellite geometry. A GNSS receiver is also assumed to remain stationary on a horizontal surface. Let the dynamical system and the linear measurement equations be as follows:

(1) $$\begin{array}{cc} x_{k} & =x_{k-1}+w_{k},\\ y_{k} & =Hx_{k}+v_{k}, \end{array}$$

(2) $$\begin{array}{cc} H & =\left(\begin{array}{ccc} -\cos\theta_{1}\sin\psi_{1} & -\cos\theta_{1}\cos\psi_{1} & -\sin\theta_{1}\\ \vdots & \vdots & \vdots \\ -\cos\theta_{5}\sin\psi_{5} & -\cos\theta_{5}\cos\psi_{5} & -\sin\theta_{5} \end{array}\right), \end{array}$$

where $x_{k}={\left(r_{e},r_{n},r_{u}\right)}^{T}$ is a position vector represented in the ENU (East-North-Up) coordinates, $y_{k}$ is a measurement vector composed of ranges from five satellites to a receiver, H is a 5×3 measurement matrix, $v_{k}$ is a measurement noise vector, and $w_{k}$ is a process noise vector. We assume that $x_{0}∼N\left(0,I\right)$, $E\left\{w_{k}\right\}=E\left\{v_{k}\right\}=0$, $E\left\{w_{k}w_{l}^{T}\right\}=Q\left(k\right)\delta_{kl}=0.01I\delta_{kl}$ (since the position vector $x_{k}$ is stationary, $w_{k}$ and Q(k) should be zero originally. However, such a choice of $w_{k}$ and Q(k) would lead to numerical problems of Kalman filter [11]. To avoid the problems, small $w_{k}$ and Q(k) are usually assumed to exist. In this paper, we suppose that “true process noise” denoted by $w_{k}$ and Q(k) includes this inevitable small noise) and $E\left\{v_{k}v_{l}^{T}\right\}=R\left(k\right)\delta_{kl}=4I\delta_{kl}$. E{a} is the expectation of a random variable *a*, and $\delta_{kl}$ is the Kronecker delta. $w_{k}$ and $v_{k}$ are produced numerically by random number. $\theta_{i}$ and $\psi_{i}$ are the elevation angle and the azimuth angle of the *i*th satellite, respectively.

**Figure 2:**
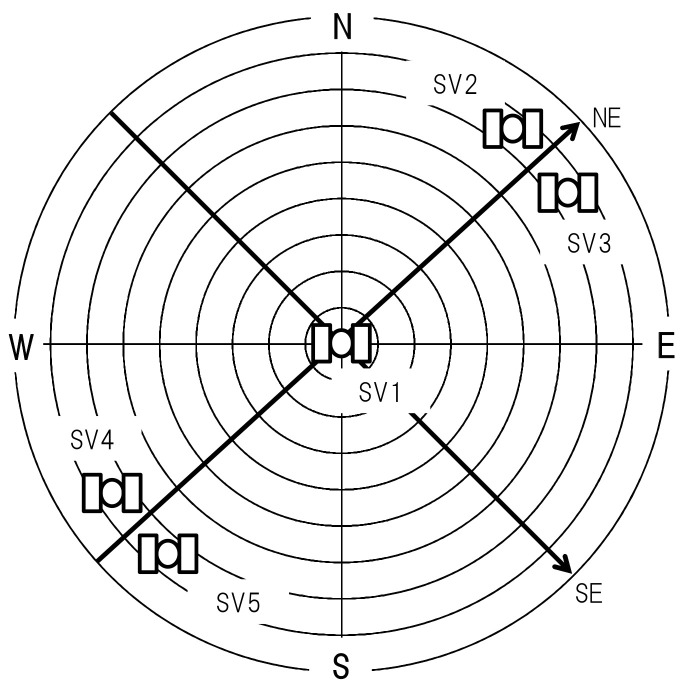
A sky plot for simulation study. SV, NE, and SE stand for Satellite Vehicle, a northeast direction, and a southeast direction respectively.

The five satellites are supposed to be located as shown in Figure 2. Their elevation angles are θ={90,15,15,15,15} [degree], and their azimuth angles are ψ={0,40,50,220,230} [degree]. The satellites are located along the northeast direction as in Figure 1. We can determine whether the satellite geometry (it is well-known that DOP (Dilution of Precision) parameters characterize satellite geometry. In this paper, we attempt to integrate satellite geometry into Kalman filter) is poor or not by evaluating the eigenvalues of $H^{T}H$; If one of eigenvalues is zero or close to zero, $H^{T}H$ is singular or almost singular, and such a satellite geometry is called poor. The eigenvectors of $H^{T}H$ corresponding to the minimum and maximum eigenvalues coincide with a southeast direction and a northeast direction in this satellite geometry (the locations of the five satellites were chosen so that the directions of the eigenvectors of $H^{T}H$ are intuitively obvious. Although this paper presents the results when the elevation angles $\theta_{i}$ for four satellites are 15 degrees (*i*= 2, … 5), similar numerical results are obtained for $\theta_{i}=0,30,$ or 45 degrees).

**Figure 3:**
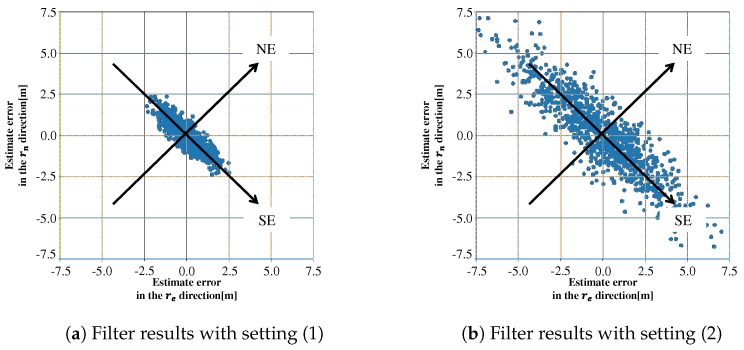
Estimated positions obtained by repeating Kalman filter computation 1000 times.

Simulation results are obtained by Kalman filter that will be described in Section 3, with two parameter settings. For setting (1), the process noise covariance $\tilde{Q}$ in Kalman filter is chosen as the true one Q. For setting (2), $\tilde{Q}$ is set by adding fictitious noise δqI to Q. Figure 3 shows the estimated positions in a $r_{e}$-$r_{n}$ plane that were obtained by repeating the filter computation 1000 times with different initial position estimates and different noise realizations.

Table 1 shows standard deviations that are computed by projecting the plotted estimate errors to two eigenvectors of $H^{T}H$, where $\sigma_{SE}$ and $\sigma_{NE}$ are standard deviations in the directions of two eigenvectors, southeast direction and northeast direction. The differences between $\sigma_{SE}$ and $\sigma_{NE}$ mainly comes from the satellite geometry in both (1) and (2). Since there is no model error in these simulations, the plotted estimation errors spread depending on the satellite geometry and the settings of process and measurement noises, and are consistent with the estimation-error covariance calculated from Kalman filter. However, we should note that the values of $\sigma_{SE}/\sigma_{NE}$ in (1) and (2) are quite different—3.48 and 4.22, respectively. This means that the estimation errors in southeast direction are spread more widely than in northeast direction even though the fictitious process noise is added equally in each direction. The fictitious noise to avoid the filter divergence makes the estimation errors worse unintentionally in the southeast direction that corresponds to the eigenvector of the smallest eigenvalue.

We devote Section 3 to reveal the theoretical relationship among the inflation of estimation-error covariance, process noise covariance, and satellite geometry. Then, in Section 4, we will describe how to reduce the unnecessary inflation of estimation-error covariance.

## 3. Sensitivity Analysis of Estimation-Error Covariance

We consider a linear system and Kalman filter for it:

(3) $$\begin{array}{cc} x_{k} & =\Phi \left(k|k-1\right)x_{k-1}+w_{k}, \end{array}$$

(4) $$\begin{array}{cc} y_{k} & =H\left(k\right)x_{k}+v_{k}, \end{array}$$

(5) $$\begin{array}{cc} \hat{x}\left(k|k-1\right) & =\Phi \left(k|k-1\right)\hat{x}\left(k-1|k-1\right), \end{array}$$

(6) $$\begin{array}{cc} P(k|k-1) & =\Phi \left(k|k-1\right)P\left(k-1|k-1\right)\Phi^{T}\left(k|k-1\right)+\tilde{Q}\left(k\right), \end{array}$$

(7) $$\begin{array}{cc} \hat{x}\left(k|k\right) & =\hat{x}\left(k|k-1\right)+K\left(k\right)\left(y_{k}-H\left(k\right)\hat{x}\left(k|k-1\right)\right), \end{array}$$

(8) $$\begin{array}{cc} P(k|k) & ={\left[P^{-1}\left(k|k-1\right)+H{\left(k\right)}^{T}{\tilde{R}}^{-1}\left(k\right)H\left(k\right)\right]}^{-1}, \end{array}$$

(9) $$\begin{array}{cc} K(k) & =P\left(k|k-1\right)H^{T}\left(k\right){\left[H\left(k\right)P\left(k|k-1\right)H^{T}\left(k\right)+\tilde{R}\left(k\right)\right]}^{-1}, \end{array}$$

where we use the same notations as in Equations (1) and (2), and variables in the filter are listed in Table 2. We suppose that $\tilde{R}\left(k\right)$ and $\tilde{Q}\left(k\right)$ are different from their true ones R(k) and Q(k).

First of all, we derive the difference equation between P(k−1|k−1) and P(k|k). Since $H^{T}\left(k\right){\tilde{R}}^{-1}\left(k\right)H\left(k\right)$ is symmetric, it can be diagonalized by an orthogonal matrix G, i.e., $H^{T}\left(k\right){\tilde{R}}^{-1}\left(k\right)H\left(k\right)=G\left(k\right)\tilde{\Lambda }\left(k\right)G^{T}\left(k\right)$, where $\tilde{\Lambda }\left(k\right)$ is a diagonal matrix whose diagonal elements are the eigenvalues of $H^{T}\left(k\right){\tilde{R}}^{-1}\left(k\right)H\left(k\right)$. Now we make the following assumptions:

**Assumption** **1.** *Φ(k)=I.*

**Assumption** **2.** *$G^{T}\left(k\right)P\left(k-1|k-1\right)G\left(k\right)$ and $G^{T}\left(k\right)\tilde{Q}\left(k\right)G\left(k\right)$ are diagonal.*

We drop the notation (k) for simplicity except for P(k−1|k−1) and P(k|k). Using Assumption 1 and substituting $H^{T}{\tilde{R}}^{-1}H=G\tilde{\Lambda }G^{T}$ into Equation (8), we can rewrite it as

(10) $$\begin{array}{cc} P(k|k) & ={\left[P^{-1}\left(k|k-1\right)+H^{T}{\tilde{R}}^{-1}H\right]}^{-1}\\ \; & =G{\left[{\left(G^{T}P\left(k-1|k-1\right)G+G^{T}\tilde{Q}G\right)}^{-1}+\tilde{\Lambda }\right]}^{-1}G^{T}. \end{array}$$

Multiplying $G^{T}$ and G to both sides of Equation (10), we obtain

(11) $$\begin{array}{c} G^{T}P\left(k|k\right)G={\left[{\left(G^{T}P\left(k-1|k-1\right)G+G^{T}\tilde{Q}G\right)}^{-1}+\tilde{\Lambda }\right]}^{-1}. \end{array}$$

From Equation (11) and Assumption 2, $G^{T}P\left(k|k\right)G$ is also diagonal. Then the iith element of $G^{T}P\left(k|k\right)G$ denoted as ${\tilde{P}}_{i,k}$ can be written as

(12) $$\begin{array}{c} {\tilde{P}}_{i,k}=\frac{{\tilde{P}}_{i,k-1}+{\tilde{q}}_{i}}{1+{\tilde{\lambda }}_{i}\left({\tilde{P}}_{i,k-1}+{\tilde{q}}_{i}\right)}, \end{array}$$

where ${\tilde{\lambda }}_{i}$ is the iith element of $\tilde{\Lambda }$, and ${\tilde{q}}_{i}$ is the iith element of $G^{T}\tilde{Q}G$. Equation (12) is the difference equation of estimation-error covariance from k−1 to *k*, and shows that ${\tilde{P}}_{i,k}$ depends on ${\tilde{q}}_{i}$ and ${\tilde{\lambda }}_{i}$, that is, process noise covariance and satellite geometry.

By using the true process noise covariance Q and a fictitious process noise δq, we represent $\tilde{Q}$ as $\tilde{Q}=Q+\delta q$. It is assumed that each component in $\tilde{Q}$ is diagonalized by G, that is, $G^{T}QG$ and $G^{T}\delta qG$ are diagonal matrices. Then, Equation (12) can be rewritten as:

(13) $$\begin{array}{c} {\tilde{P}}_{i,k}=\frac{{\tilde{P}}_{i,k-1}+q_{i}+\delta q_{i}}{1+{\tilde{\lambda }}_{i}\left({\tilde{P}}_{i,k-1}+q_{i}+\delta q_{i}\right)}, \end{array}$$

where $q_{i}$ and $\delta q_{i}$ are the iith element of $G^{T}QG$ and $G^{T}\delta qG$, respectively. Now we define the variation of ${\tilde{P}}_{i,k}$ due to $\delta q_{i}$ as $\Delta P_{i}$. From Equation (13), $\Delta P_{i}$ is approximated as the following equation.

(14) $$\begin{array}{cc} \Delta P_{i} & \approx {\left.\frac{\partial {\tilde{P}}_{i,k}}{\partial \delta q_{i}}\right|}_{\delta q_{i}=0}\delta q_{i}\\ \; & =\frac{1}{{\left\{1+{\tilde{\lambda }}_{i}\left({\tilde{P}}_{i,k-1}+q_{i}\right)\right\}}^{2}}\delta q_{i}. \end{array}$$

From Equation (14), we examine the sensitivity of $\Delta P_{i}$ with respect to ${\tilde{\lambda }}_{i}$. Since $H^{T}{\tilde{R}}^{-1}H$ is a positive semidefinite matrix, ${\tilde{\lambda }}_{i}\geq 0$ and

(15) $$\begin{array}{c} \frac{\partial \Delta P_{i}}{\partial {\tilde{\lambda }}_{i}}<0,\;\mathrm{for}\;\delta q_{i}>0. \end{array}$$

That is, $\Delta P_{i}\left({\tilde{\lambda }}_{i}\right)$ decreases monotonically as ${\tilde{\lambda }}_{i}$ goes from zero to infinity. At the limits ${\tilde{\lambda }}_{i}\to +0$ and ${\tilde{\lambda }}_{i}\to \infty$, the values of $\Delta P_{i}\left({\tilde{\lambda }}_{i}\right)$ are given as

$$\begin{array}{c} \lim_{{\tilde{\lambda }}_{i}\to 0}\Delta P_{i}=\delta q_{i},\;\;\lim_{{\tilde{\lambda }}_{i}\to \infty }\Delta P_{i}=0. \end{array}$$

For good satellite geometry, there is no eigenvalue ${\tilde{\lambda }}_{i}$ close to zero. The inflations of ${\tilde{P}}_{i,k}$ that are caused by the fictitious noise $\delta q_{i}$ between time steps k−1 and *k* are suppressed for all *i* by the observation at time step *k*, according to Equation (14). By choosing an appropriate value of $\delta q_{i}$ through trial and error, we can keep the covariance P(k|k) reasonably large to avoid the filter divergence even for the system with model errors. On the other hand, at least one of eigenvalues is zero or close to zero for poor satellite geometry. If we choose the size of $\delta q_{i}$ for good satellite geometry, it may be too large for poor satellite geometry and cause unintentionally large inflation of ${\tilde{P}}_{i,k}$. Too large covariance ${\tilde{P}}_{i,k}$ leads to widely distributed estimation errors along the corresponding direction and degrades the filter performance.

In the next section, we will propose a new way to choose the fictitious process noise $\delta q_{i}$ based on satellite geometry $H^{T}\left(k\right)H\left(k\right)$ to reduce the inflation. To derive the choice, we make an additional assumption:

**Assumption** **3.** *$\tilde{R}\left(k\right)=r_{k}I$, where $r_{k}$ is a positive scalar.*

From Assumption 3, $H^{T}\left(k\right){\tilde{R}}^{-1}\left(k\right)H\left(k\right)$ can be rewritten as

$$\begin{array}{c} H^{T}\left(k\right){\tilde{R}}^{-1}\left(k\right)H\left(k\right)=\frac{1}{r_{k}}G\left(k\right)\Lambda \left(k\right)G^{T}\left(k\right), \end{array}$$

where Λ(k) is diagonal. From Equations (13) and (14), we obtain

(16) $$\begin{array}{cc} {\tilde{P}}_{i,k} & =\frac{r_{k}\left({\tilde{P}}_{i,k-1}+q_{i}+\delta q_{i}\right)}{r_{k}+\lambda_{i}\left({\tilde{P}}_{i,k-1}+q_{i}+\delta q_{i}\right)}, \end{array}$$

(17) $$\begin{array}{cc} \Delta P_{i} & \approx \frac{r_{k}^{2}}{{\left(r_{k}+\lambda_{i}\left({\tilde{P}}_{i,k-1}+q_{i}\right)\right)}^{2}}\delta q_{i}, \end{array}$$

where $\lambda_{i}$ is the iith element of Λ(k). It should be noted that $\lambda_{i}$ is an eigenvalue of $H^{T}\left(k\right)H\left(k\right)$ and, unlike ${\tilde{\lambda }}_{i}$, represents the satellite geometry independently of $\tilde{R}\left(k\right)$.

Moreover, we can compute a steady-state solution of Equation (12) (or Equation (16)), under the assumptions that $\tilde{R}\left(k\right)$, H(k) and $\tilde{Q}\left(k\right)$ are time-invariant, and $G^{T}P\left(0|0\right)G$ is diagonal. Denoting the steady values of ${\tilde{P}}_{i,k}$ and ${\tilde{P}}_{i,k-1}$ as ${\tilde{P}}_{i}$ and substituting them into Equation (12), we can obtain a steady-state solution:

(18) $$\begin{array}{c} {\tilde{P}}_{i}=\frac{-{\tilde{q}}_{i}+\sqrt{{\tilde{q}}_{i}^{2}+\frac{4{\tilde{q}}_{i}}{{\tilde{\lambda }}_{i}}}}{2}. \end{array}$$

The solution in Equation (18) is equivalent to the solution of an algebraic Riccati equation of estimation-error covariance [22]. It should be noted that the solution in Equation (18) is consistent with the results in Section 2.2. The values of ${\tilde{P}}_{i}$ that are calculated with ${\tilde{\lambda }}_{i}$ and ${\tilde{q}}_{i}$ for the motivating example coincide with the standard deviations in Table 1. Even though a steady-state solution is well-known, the analysis in this section revealed how the covariance ${\tilde{P}}_{i,k}$ is inflated at each time step depending on the fictitious noise $\delta q_{i}$ and the satellite geometry $\lambda_{i}$ (or ${\tilde{\lambda }}_{i}$). By using the analytical result in Equation (17), we will propose a novel process noise model in the next section to avoid an unintentionally large inflation at each time step.

## 4. Novel Process Noise Model for GNSS Stationary Positioning

### 4.1. Extended Kalman Filter

In this section, we describe the extended Kalman filter for GNSS stationary positioning. Since the pseudo-range and Doppler frequency measurement equations are non-linear, the extended Kalman filter (referred as EKF) is generally used. We define the state vector as $x={\left[r^{T},t_{s}^{T},t_{b},{\dot{t}}_{b}\right]}^{T}$, where r[m] is the position vector of a receiver, $t_{b}$[m] is a clock bias, and ${\dot{t}}_{b}$[m/s] is a clock drift. $t_{s}$[m] is an inter-system bias (ISB) vector between GPS time and other GNSS’s time. We suppose to use a multi-GNSS single frequency receiver, for example, FURUNO GN-8720, and $t_{s}$ consists of three elements that are GPS-QZSS, GPS-GLONASS, and GPS-Galileo ISBs.

The dynamical system and the measurement equations are given as follows:

(19) $$\begin{array}{cc} x_{k} & =\Phi \left(k|k-1\right)x_{k-1}+w_{k}, \end{array}$$

(20) $$\begin{array}{cc} y_{k} & =h\left(x_{k}\right)+v_{k}, \end{array}$$

(21) $$\begin{array}{cc} h^{\left(j\right)}\left(x_{k}\right) & =||r\left(k\right)-r^{\left(j\right)}\left(k\right)\left|\right|+t_{b}+t_{m}, \end{array}$$

(22) $$\begin{array}{cc} h^{\left(j+M\right)}\left(x_{k}\right)\; & =-\frac{{\left(r\left(k\right)-r^{\left(j\right)}\left(k\right)\right)}^{T}{\dot{r}}^{\left(j\right)}\left(k\right)}{||r\left(k\right)-r^{\left(j\right)}\left(k\right)\left|\right|}+{\dot{t}}_{b}, \end{array}$$

where

(23) $$\begin{array}{cc} \; & \Phi \left(k+1|k\right)=\left[\begin{array}{cc} I_{6\times 6} & O_{6\times 2}\\ O_{2\times 6} & F \end{array}\right],F=\left[\begin{array}{cc} 1 & \Delta t\\ 0 & 1 \end{array}\right], \end{array}$$

$h^{\left(j\right)}\left(x_{k}\right)$ and $h^{\left(j+M\right)}\left(x_{k}\right)$ are the *j*th and the j+Mth elements of $h(x_{k})$, $r^{\left(j\right)}\left(k\right)$ and ${\dot{r}}^{\left(j\right)}\left(k\right)$ are the position and velocity vectors of the *j*th satellite, and $t_{m}$ is the ISB of the GNSS system to which the *j*th satellite belongs. *M* is the number of satellites.

The EKF for this system consists of the computations outlined from Equation (5) to Equation (9), but with Equation (7) replaced by

(24) $$\begin{array}{cc} \hat{x}\left(k|k\right) & =\hat{x}\left(k|k-1\right)+K\left(k\right)\left[y_{k}-h\left(\hat{x}\left(k|k-1\right)\right)\right], \end{array}$$

and H(k) computed as

$$\begin{array}{c} H\left(k\right)={\left.\frac{\partial h(x_{k})}{\partial x_{k}}\right|}_{x_{k}=\hat{x}\left(k|k-1\right)}. \end{array}$$

The EKF may often produce a biased estimate and diverge due to unmodeled measurement errors, dynamics errors, and non-linear effects of Equation (21) [23]. To cover the uncertainties by a fictitious process noise, we usually choose the process noise covariance in the EKF as

(25) $$\begin{array}{c} \tilde{Q}=Q+\delta q. \end{array}$$

For the EKF in this paper, we set Q to 0.01I to avoid the numerical problems as in Section 2.2. This $\tilde{Q}$ makes gain matrix K(k) large from Equations (6) and (9), thus the EKF updates the estimate $\hat{x}$ by overweighting current observations. It should be noted that a larger $\tilde{R}$ cannot be effective in preventing filter divergence, since a larger $\tilde{R}$ makes K(k) smaller. We choose $\tilde{R}$ so that it represents actual measurement noise level. In this paper, $\tilde{R}$ is determined based on the Signal-Noise-Ratios (SNRs) of the receiver FURUNO GN-8720 as follows [2]:

(26) $$\begin{array}{cc} \tilde{R} & =\mathrm{diag}(\sigma_{1}^{2}\left(s_{1}\right),\ldots ,\sigma_{M}^{2}\left(s_{M}\right),\mu_{1}^{2}\left(s_{1}\right),\ldots ,\mu_{M}^{2}\left(s_{M}\right)),\\ \sigma_{k} & =0.64+784e^{-0.142s_{k}},\\ \mu_{k} & =0.0125+6767e^{-0.267s_{k}},\;\;k=1,\ldots ,M, \end{array}$$

where $s_{k}$, $\sigma_{k}$ and $\mu_{k}$ are the SNR, the standard deviations of pseudo-range noise and Doppler frequency noise for the *k*th satellite signal.

### 4.2. Process Noise Covariance Based on Satellite Geometry

In practical applications of EKF, it is not easy to find an appropriate choice of the fictitious noise δq in Equation (25). It is often chosen and fixed through a trial and error process, so that a required performance of EKF is obtained. If the satellite geometry is always good and all the eigenvalues $\lambda_{i}$ are equally large, such a choice of δq could work well. In this paper, we call it the conventional choice, and consider only δq in the following form:

(27) $$\begin{array}{c} \delta q=\delta qI, \end{array}$$

where δq is a positive scalar. However, if the satellite geometry is poor, the conventional choice of δq would cause an unintentional inflation of P as described in Section 2 and Section 3.

In this section, we propose a novel way to choose the fictitious noise δq based on satellite geometry by assuming that Equation (16) approximately holds in our EKF. The basic idea is to choose δq so that $\Delta P_{i}$ in Equation (17) is less than or equal to a specified value $c_{i}$ at each time step *k* in order to suppress the unintentional inflation. The fictitious noise is chosen as follows.

(28) $$\begin{array}{cc} \delta q & =G\left(k\right)QG^{T}\left(k\right),\\ Q_{i} & =\left\{\begin{array}{cc} \delta q_{i}^{\prime } & \mathrm{if}\;\delta q_{i}^{\prime }\leq \delta q\\ \delta q & \mathrm{else} \end{array}\right.,\\ \delta q_{i}^{\prime } & =\frac{{\left(r_{k}+\lambda_{i}\left({\tilde{P}}_{i,k-1}+q_{i}\right)\right)}^{2}}{r_{k}^{2}}c_{i}, \end{array}$$

where the matrix Q is diagonal, and $Q_{i}$ is the *i*th diagonal element of Q, and δq is a positive scalar for the conventional choice. From Equations (17) and (28), if $\delta q_{i}^{\prime }\leq \delta q$, $\Delta P_{i}$ satisfies $\Delta P_{i}\approx c_{i}$ even for a small $\lambda_{i}$. If $\delta q_{i}^{\prime }>\delta q$, $\Delta P_{i}<c_{i}$ holds approximately even for $\delta q_{i}=\delta q$. Consequently, we can expect that the unintentional inflation of P is suppressed, because $\Delta P_{i}$ is limited to the maximum value of $c_{i}$, regardless of satellite geometry $\lambda_{i}$. For the proposed choice of δq, we can keep an appropriate inflation of P by the parameters $c_{i}$. We will determine $c_{i}$ through simulation study in Section 5.

Moreover, the steady-state solution ${\tilde{P}}_{i}$ in Equation (18) does not hold for δq in Equation (28), because ${\tilde{q}}_{i}$ in Equation (12) includes ${\tilde{P}}_{i,k-1}$. By substituting Equation (28) into Equation (16), we can obtain the following equation:

(29) $$\begin{array}{cc} {\tilde{\lambda }}_{i}^{3}c_{i}{\tilde{P}}_{i}^{3}+\left({\tilde{\lambda }}_{i}+{\tilde{\lambda }}_{i}^{2}c_{i}+2{\tilde{\lambda }}_{i}^{3}c_{i}q_{i}\right){\tilde{P}}_{i}^{2}+ & \left({\tilde{\lambda }}_{i}q_{i}-{\tilde{\lambda }}_{i}c_{i}+{\tilde{\lambda }}_{i}^{3}c_{i}q_{i}^{2}\right){\tilde{P}}_{i}\\ - & (c_{i}+q_{i}+2{\tilde{\lambda }}_{i}c_{i}q_{i}+{\tilde{\lambda }}_{i}^{2}c_{i}q_{i}^{2})=0. \end{array}$$

By solving the above equation numerically, we can obtain an approximate steady-state solution ${\tilde{P}}_{i}$.

Strictly speaking, Equation (16) does not hold for the EKF in this section, because the three assumptions in Section 3 may be unsatisfied, that is to say, (1) Φ(k)≠I, (2) $G^{T}\left(k\right)P\left(k-1|k-1\right)G\left(k\right)$ is not diagonal, and (3) $\tilde{R}\neq r_{k}I$. For Assumption 3, we choose $\tilde{R}\left(k\right)$ as diagonal and give its diagonal elements based on SNRs in this paper. Since SNRs of LOS signals usually have similar values, we can expect that $\tilde{R}\left(k\right)\approx r_{k}I$ and Assumption 3 is approximately satisfied. For Assumptions 1 and 2, we cannot justify them theoretically. Although Assumption 1 is necessary to show that both P(k|k) and P(k−1|k−1) are diagonalized by G(k), they are almost diagonalized for the experimental results shown in Section 6. Therefore, Assumption 2 is approximately satisfied in the experimental results, and then we do not need Assumption 1 to show it. However, for Φ≠I, Equation (11) itself does not hold. In this paper, we suppose that the effects of Φ with F in Equation (23) are small, because only one nondiagonal element is nonzero. We also note that, in Assumption 2, $\tilde{Q}$ can be chosen with δq in Equation (28) such that $G^{T}\left(k\right)\tilde{Q}G\left(k\right)$ is diagonal. As a result, we believe that Equation (16) approximately holds in our EKF.

We finally summarize the proposed noise model. Employing the fictitious noise δq in Equation (28), $\tilde{Q}$ varies depending on satellite geometry, and $\Delta {\tilde{P}}_{i}$ can be insensitive to satellite geometry. We, therefore, call the model *satellite-geometry adaptive model*. Although a noise-adaptive model introduced in Section 1 is well known, $\tilde{Q}$ is estimated based on recent observations, and an accurate estimation would be difficult for poor satellite geometry. To our best knowledge, our model is the first process noise model that changes with $H^{T}H$, i.e., satellite geometry.

## 5. Simulation Study

We present here some simulation results of the proposed process noise model to demonstrate its effectiveness and to determine an appropriate value of $c_{i}$. The model is applied to the Kalman filter for the simplified system in Section 2.2, where all the assumptions in Section 3 are satisfied and Equation (16) holds.

We consider the following three settings of the process noise $\tilde{Q}$.

Setting (1): No fictitious process noise:

(30) $$\begin{array}{c} \tilde{Q}=Q \end{array}$$

Setting (2): Conventional choice of fictitious process noise:

(31) $$\begin{array}{c} \tilde{Q}=Q+\delta qI \end{array}$$

Setting (3): Proposed choice of fictitious process noise:

(32) $$\begin{array}{c} \tilde{Q}=Q+G\left(k\right)QG^{T}\left(k\right) \end{array}$$

The first and second settings are the same as the settings in Figure 3a,b, where δq is chosen as δq=1 in Equation (31). In this paper, we assume that, for Equation (32), all the $c_{i}$ are the same, that is, $c_{i}=c$ for ∀i. Numerical simulations for setting (3) were performed with different values of *c*: (3-a) with c=1.0 to (3-f) with c=0.01 as shown in Table 3. The standard deviations $\sigma_{SE}$, $\sigma_{NE}$, and their ratios for all the settings are summarized in Table 3, where the results for settings (1) and (2) in Table 1 are shown again. The Root Mean Squared Error (RMSE) *d* is also computed for the three-dimensional position to show the positioning accuracy in the table.

For determining an appropriate value of *c*, we should pay attention to two points: avoiding the filter divergence and suppressing the unintentional inflation of P along the eigenvectors corresponding to small eigenvalues $\lambda_{i}$. For the first point, $\sigma_{NE}$, that is the standard deviation along the direction with a large eigenvalue, is increased to 0.80 in setting (2), comparing with setting (1). A similar magnitude of $\sigma_{NE}$ would be necessary even in setting (3) for the divergence avoidance. For the second point, we check the ratio of $\sigma_{SE}$ to $\sigma_{NE}$. In setting (1), the ratio comes only from the satellite geometry, because there is no model error or fictitious noise. Therefore, we suppose that the ratio in setting (1) is desirable even in setting (3) for suppressing the inflation. From these two points, we can choose setting (3-c) c=0.36 for the proposed noise covariance model in this paper. Moreover, it should be noted that the values of standard deviations in (3-a) to (3-f) are consistent with the steady-state solutions computed from Equation (29).

**Figure 4:**
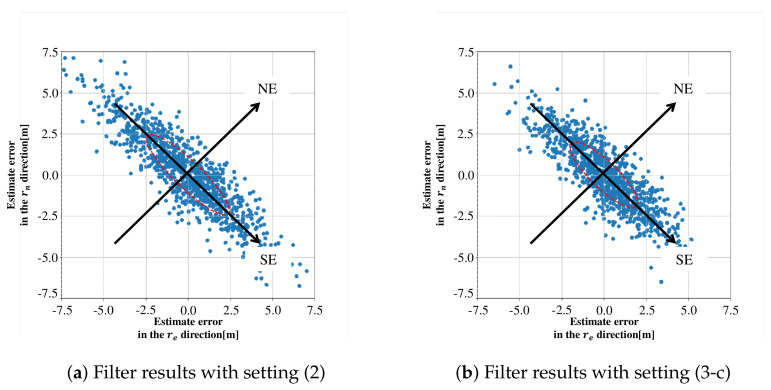
Estimated positions obtained for settings (2) and (3-c).

The effectiveness of the proposed noise model is verified by comparing the simulation results in settings (2) and (3-c). The estimated positions in a $r_{e}$-$r_{n}$ plane for the two settings are plotted in Figure 4a,b, where the filter computation was repeated 1000 times for each setting as in Section 2.2 and Figure 4a is the same as Figure 3b. Ellipsoids in these figures are the 1σ contours of probability density function (Gaussian) that are calculated from Equation (18) for Figure 4a and from Equation (29) for Figure 4b. The distribution of horizontal position errors obtained by numerical simulations coincides with the ellipsoid obtained theoretically for each setting. We can see that the estimation errors in setting (3-c) are reduced in the direction of southeast, that is, the direction of the minimum eigenvalue of $H^{T}H$. In this numerical results, the proposed noise model suppresses the unintentional inflation of $\sigma_{SE}$ by about 20%. It should be noted that these results show that both precision and accuracy of positioning in the horizontal plane are improved in setting (3-c), because the mean value of estimation errors is almost zero. We can also check the positioning accuracy that includes the vertical errors, by comparing the RMSEs for settings (2) and (3-c) in Table 3.

## 6. Experimental Results of Stationary Positioning

**Figure 5:**
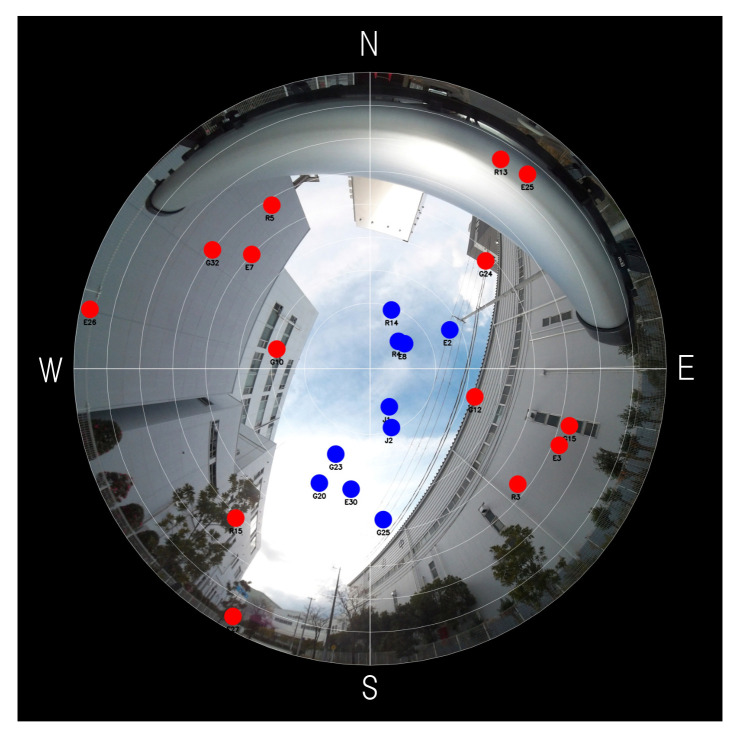
A fisheye-camera image taken at the place where we gathered data.

This section presents experimental results to show the effectiveness of the proposed process noise model by applying it to actual data obtained in an urban canyon. The data was collected at the time and location shown in Table 4, and Figure 5 is a fisheye-camera image with a satellite sky plot at the beginning of data collection. We used a multi-GNSS single frequency receiver, FURUNO GN-8720, that can receive the signals of GPS, QZSS L1C/A, GLONASS L1OF, and Galileo E1, and acquired about ten minutes the data that includes pseudo-ranges, Doppler frequencies, navigation messages, and so on. The receiver was fixed at the location that corresponds to the center of Figure 5 during the experiment, and the pseudo-ranges and Doppler frequencies were used as the measurements for EKF. In the figure, blue dots are LOS satellites, and red dots are NLOS satellites, as in Section 2.1. By detecting NLOS satellites from the fisheye-camera images, the signals from them were excluded from the measurements. The LOS satellites are quite close to each other, and the satellite geometry can be considered as poor, because the minimum eigenvalue of the LOS satellite geometry is always about 0.01 in the data (although multipath signals may occur via diffraction/reflection even for LOS satellites, they would be largely attenuated compared to LOS direct signals. In this paper, we do not consider the effects of multipath signals by assuming that they are sufficiently small).

Based on the numerical results shown in Section 5, we examine positioning performance of the EKF in Section 4.1 with two parameter settings for process noise covariance: Setting (2) with δq=1 and Setting (3-c) with c=0.36. The EKFs with the conventional and the proposed noise settings were implemented in a laptop computer as post-processing programs, by customizing the EKF program implemented in FURUNO GN-8720. The EKFs compute the estimated position of the receiver by using only the measurements from LOS satellites.

In order to obtain an ensemble average of estimation errors, we make *N* measurement data sets whose length is *l*, from the data acquired for about ten minutes, by shifting the start point by one step. The EKF calculation is performed for each of the *N* data sets, $Y_{1}=\left\{y_{1},y_{2},\cdots ,y_{l}\right\},Y_{2}=\left\{y_{2},y_{3},\cdots ,y_{l+1}\right\},\cdots ,Y_{N}=\left\{y_{N},y_{N+1},\cdots ,y_{l+N-1}\right\}$, with the initial conditions shown in Table 4. The length *l* of each data set is chosen such that the changes in $\hat{x}$ and P become sufficiently small in the EKF as the time step *k* approaches *l*. We focus on the estimation error in position at the final step, $\hat{r}\left(l|l\right)-r$, where the true position r is known as in Table 4, and denote it as ${\tilde{r}}_{m}$ for *m*th data set.

We can compute estimation-error covariance Σ and RMSE (Root Mean Squared Error) *d* as follows:

(33) $$\begin{array}{cc} \Sigma & =\frac{1}{N-1}\sum_{m=1}^{N}\left({\tilde{r}}_{m}-\bar{r}\right){\left({\tilde{r}}_{m}-\bar{r}\right)}^{T},\\ \bar{r} & =\frac{1}{N}\sum_{m=1}^{N}{\tilde{r}}_{m}, \end{array}$$

(34) $$\begin{array}{cc} d & =\sqrt{\frac{1}{N}\sum_{m=1}^{N}{\tilde{r}}_{m}^{T}{\tilde{r}}_{m}}. \end{array}$$

**Figure 6:**
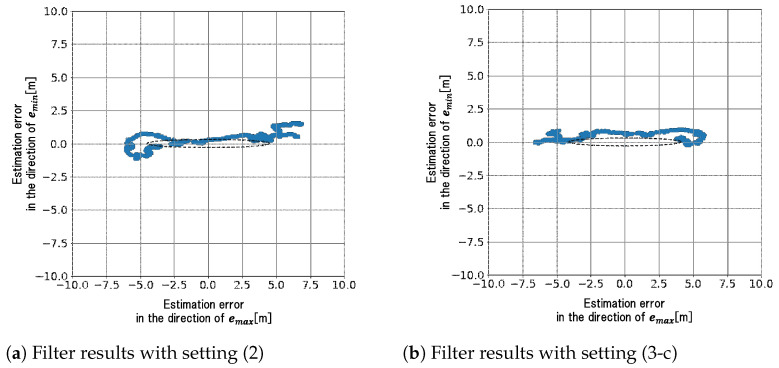
Estimated position errors in the plane spanned by $e_{min}$ and $e_{max}$.

The minimum and maximum standard deviations $\sigma_{min}$ and $\sigma_{max}$ of the estimation errors ${\tilde{r}}_{m}$ are obtained by calculating the minimum and maximum eigenvalues of Σ. Table 5 summarizes the values of $\sigma_{min}$, $\sigma_{max}$, $\sigma_{max}/\sigma_{min}$ and *d* for the two settings. The following two points can be seen in Table 5: (a) $\sigma_{max}$ and $\sigma_{max}/\sigma_{min}$ in setting (3-c) are less than the ones in setting (2), while the values of $\sigma_{min}$ in both settings are almost the same, and (b) RMSE *d* in setting (3-c) is also less than the one in setting (2). The first point (a) would indicate that the proposed noise model can suppress the unintentional inflation of estimation-error covariance even for actual data in an urban canyon. From the second point (b), the positioning accuracy with the proposed noise model is also improved compared to the conventional noise model. The estimation errors ${\tilde{r}}_{m}$ for the two settings are shown in Figure 6a,b, where the errors ${\tilde{r}}_{m}$ are projected to the plane spanned by the unit eigenvectors $e_{min}$ and $e_{max}$ that correspond to $\sigma_{min}$ and $\sigma_{max}$, respectively. The ellipsoid in each figure is the 1σ contour of probability density function that is supposed to be Gaussian with the standard deviations $\sigma_{min}$ and $\sigma_{max}$ in Table 5.

Although the results in this section demonstrate that the suppression of covariance inflation is achieved by the proposed noise model, it should be noted that the eigenvector corresponding to $\sigma_{max}$ is almost along the altitude direction. The standard deviations $\sigma_{min}$ and $\sigma_{max}$ in Table 5 are also quite different from the ones calculated from Equations (18) and (29). These differences from the simulation results in Section 5 may be due to the following two reasons. First, the satellite geometry in Figure 5 is largely different from the one for the simulation results, because there is no LOS satellite with a low elevation angle in Figure 5. The uncertainty in the altitude direction would be large due to the clock bias $t_{b}$ for such a satellite geometry (if the elevation angles of all the satellites are close to 90 degrees, the errors in altitude estimation and clock bias estimation would be indistinguishable. Through numerical simulations based on the simplified simulation model introduced in Section 2.2 for the same satellite geometry as in Figure 5, we can see that the uncertainty in the altitude direction with clock bias estimation is much larger than the one without it). Secondly, Assumption 1 is not satisfied in the EKF in Section 4.1. Equations (18) and (29) do not hold without the assumption. The non-diagonal component of F in Equation (23) would be non-negligible, especially because the position estimation in the altitude direction is highly dependent on the clock bias $t_{b}$.

The above inconsistency between the simulation results and the experimental results could be resolved by extending the proposed process noise model to the systems without the assumptions in Section 3. Our preliminary results indicate that theoretical extension of the sensitivity analysis is possible, and it will allow us to develop a process noise model in a more consistent manner for the systems without the assumptions. Further improvement of the estimation performance may also be possible by changing the choice of *c* or δq itself. The extension and improvement will be presented in our next paper.

## 7. Conclusions

This paper pointed out that a fictitious process noise to prevent the filter divergence can degrade the filter performance for a poor satellite geometry due to an unintentional inflation of estimation-error covariance. A sensitivity analysis of estimation-error covariance by fictitious process noise and satellite geometry was performed under some assumptions, and we proposed a novel model of the fictitious process noise based on satellite geometry in order to suppress the unintentional inflation of estimation-error covariance. The effectiveness of the proposed noise model was shown via simulation and experimental results. Although the sensitivity analysis and the proposed model derived from it are based on the assumptions, the approach in this paper can be extended to the systems without the assumptions. The extension and further improvement of the proposed approach will be presented in our future work.

## Figures and Tables

**Table 1:** Standard deviations of estimate errors.

Settings	(1)	(2)
$\sigma_{SE}$[m]	1.08	3.38
$\sigma_{NE}$[m]	0.31	0.80
$\sigma_{SE}/\sigma_{NE}$	3.48	4.22

**Table 2:** Filter variables.

Variable	Definition	
$\hat{x}\left(k|k-1\right)$	Prior estimate	
$\hat{x}\left(k|k\right)$	Posterior estimate	
P(k|k−1)	Prior estimation-error covaraiance	
P(k|k)	Posterior estimation-error covaraiance	
Φ(k|k−1)	State transition matrix	
$y_{k}$	Measurement vector	
H(k)	Measurement matrix	
$\tilde{R}\left(k\right)$	Measurement noise covariance	
$\tilde{Q}\left(k\right)$	Process noise covariance	
K(k)	Filter gain matrix	
*k*	Time step	

**Table 3:** Standard deviations, their ratios, and RMSEs with different process noise settings.

Settings	(1)	(2)	(3-a)	(3-b)	(3-c)	(3-d)	(3-e)	(3-f)
$\sigma_{SE}$ [m]	1.08	3.38	3.52	3.14	2.71	2.22	1.63	1.29
$\sigma_{NE}$ [m]	0.31	0.80	0.92	0.87	0.80	0.68	0.49	0.38
$\sigma_{SE}/\sigma_{NE}$	3.48	4.22	3.82	3.61	3.39	3.26	3.32	3.39
*d*	1.21	3.65	3.83	3.45	3.01	2.49	1.84	1.46

The settings from (3-a) to (3-f) are with *c* = {1.0, 0.64, 0.36, 0.16, 0.04, 0.01}.

**Table 4:** Algorithm and experimental settings.

Configurations and Parameters		Remarks
True location	34.713817 N	Latitude [degree]
	135.335599 E	Longitude [degree]
	40.49	Ellipsoidal height [m]
Date of experiment	18/12/2020 04:15:37–04:26:20	UTC
Antenna	Taoglass AA171.301111	
δq	1	Equation (31)
*c*	0.36	Equations (28) and (32)
$\hat{x}\left(0|0\right)$	$\hat{x}\left(0|0\right)∼N\left(x_{true}\left(0\right),100I\right)$	$x_{true}\left(0\right)$ is the true state of $x_{0}$
P(0|0)	100I	Initial value of P
*l*	120	Length of each data set
*N*	534	Number of data sets

**Table 5:** Standard deviations from estimation-error covariance Σ, their ratios, and RMSEs.

Settings	(2)	(3-c)
$\sigma_{max}$ [m]	4.50	4.11
$\sigma_{min}$ [m]	0.31	0.30
$\sigma_{max}/\sigma_{min}$	14.33	13.57
*d*	4.90	4.44

## Data Availability

The data presented in this study are partially available on request from the corresponding author.

## Abbreviations

The following abbreviations are used in this manuscript:

GNSS	Global Navigation Satellite System
NLOS	Non-Line-Of-Sight
LOS	Line-Of-Sight
EKF	Extended Kalman Filter
SNR	Signal to Noise Ratio
RMSE	Root Mean Squared Error

## References

[1] Groves P.D., Jiang Z., Rudi M., Strode P. A Portfolio Approach to NLOS and Multipath Mitigation in Dense Urban Areas. Proceedings of the ION GNSS+ 2013.

[2] Tominaga T., Kubo N. (2019). Performance Assessment of Adaptive Kalman Filter-Based GNSS PVT and Integrity in Dense Urban Environment. Trans. Navig..

[3] Berman Z. Outliers Rejection in Kalman Filtering—Some New Observations. Proceedings of the IEEE/ION Position, Location and Navigation Symposium—PLANS 2014.

[4] Groves P.D. (2007). Principles of GNSS, Inertial, and Multisensor Integrated Navigation Systems (GNSS Technology and Applications).

[5] Castaldo G., Angrisano A., Gaglione S., Troisi S. (2014). P-RANSAC: An Integrity Monitoring Approach for GNSS Signal Degraded Scenario. Int. J. Navig. Observ..

[6] Kato S., Kitamura M., Suzuki T., Amano Y. (2016). NLOS Satellite Detection Using a Fisheye Camera for Improving GNSS Positioning Accuracy in Urban Area. J. Robot. Mechatr..

[7] Suzuki T., Kubo N. NLOS GNSS Signal Detection Using Fish-eye Camera for Vehicle Navigation in Urban Environments. Proceedings of the ION GNSS+ 2015.

[8] Kbayer N., Sahmoudi M., Chaumette E. Robust GNSS Navigation in Urban Environments by Bounding NLOS Bias of GNSS Pseudoranges Using a 3D City Model. Proceedings of the ION GNSS+ 2015.

[9] Wen W., Zhang G., Hsu L. (2019). Correcting NLOS by 3D LiDAR and Building Height to Improve GNSS Single Point Positioning. J. Inst. Navig..

[10] Kalman R.E. (1960). A New Approach to Linear Filtering and Prediction Problems. Trans. ASME.

[11] Parkinson B.W., Spliker J.J. (1996). Global Positioning System: Theory and Applications.

[12] Fitzgerald R. (1971). Divergence of the Kalman Filter. IEEE Trans. Autom. Control.

[13] Jazwinski A.H. (1970). Stochastic Processes and Filtering Theory.

[14] Gelb A. (1974). Applied Optimal Solutions.

[15] Shi L., Johansson K.H., Murray R.M. Kalman Filtering with Uncertain Process and Measurement Noise Covariances with Application to State Estimation in Sensor Networks. Proceedings of the IEEE International Conf. on Control Applications.

[16] Simon D. (2006). Optimal State Estimation.

[17] Ding W., Wang J., Rizos C., Kinlyside D. (2007). Improving Adaptive Kalman Estimation in GPS/INS Integration. J. Navig..

[18] Hu C., Chen W., Chen Y., Dajie L. (2003). Adaptive Kalman Filtering for Vehicle Navigation. J. Glob. Position. Syst..

[19] Mehra R., Dajie L. (1970). On the Identification of Variances and Adaptive Kalman Filtering. IEEE Trans. Autom. Control.

[20] Zhang J., Kaess M., Singh S. On Degeneracy of Optimization-based State Estimation Problems. Proceedings of the IEEE International Conference on Robotics and Automation.

[21] Simon D., Chia T.L. (2002). Kalman Filtering with State Equality Constraints. IEEE Trans. Aerosp. Electron. Syst..

[22] Ammar G.S., Benner P., Mehrmann V. (1993). A Multishift Algorithm for the Numerical Solution of the Algebraic Riccati Equation. Electron. Trans. Numer. Anal..

[23] Moorman M.J., Bullock T.E. Mathematical Analysis of Bias in the Extended Kalman Filter. Proceedings of the 30th IEEE Conference on Decision and Control.

